# Emerging technologies for the management of diabetic foot ulceration: a review

**DOI:** 10.3389/fcdhc.2024.1440209

**Published:** 2024-11-12

**Authors:** Ajaytaj Singh Sidhu, Viktoriia Harbuzova

**Affiliations:** ^1^ I Horbachevsky Ternopil National Medical University, Ternopil, Ukraine; ^2^ Faculty of Natural Sciences, Sumy State University, Sumy, Ukraine

**Keywords:** diabetics, foot ulcer, artificial intelligence, risk methodology, medication, technologies

## Abstract

Diabetic foot ulcers (DFUs) and infections are common complications that frequently result in reduced quality of life and even morbidity for patients with diabetes. This paper highlights significant findings in DFU treatments and emerging advanced technologies for monitoring ulceration in patients with diabetes. The management of DFUs requires a multidisciplinary approach that involves patient education. It is well-established that poor glycemic control significantly contributes to diabetic foot ulcer complications, presenting global challenges in quality of life, economics, and resource allocation, affecting approximately half a billion people and potentially leading to lower limb amputation or mortality. Therefore, effective DFU management necessitates a multidisciplinary approach that includes patient education. However, current clinical guidelines for DFU treatment are not performing effectively, resulting in unnecessary increases in financial and emotional burden on patients. Researchers have experimented with advanced technologies and methods, including traditional approaches, to address complications related to DFU healing. This paper also presents the evolution of patents in the field of DFU medication and advanced diagnostic methods, showcasing relevant innovations that may benefit a wide range of researchers.

## Graphical Abstract

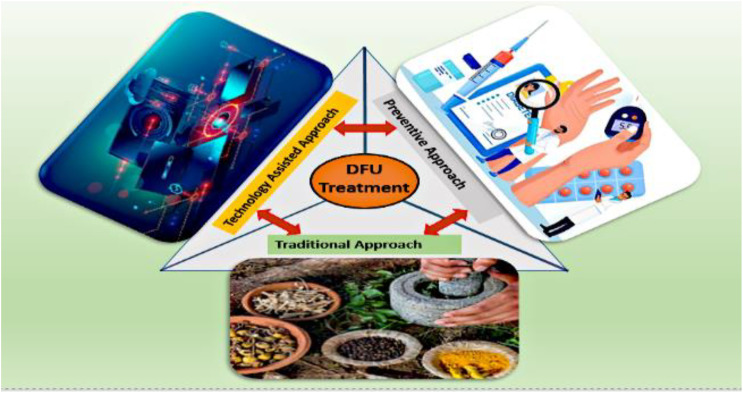

## 1 Introduction

Diabetic foot ulcers (DFUs) are a significant complication associated with diabetes mellitus, characterized by infection, ulceration, or tissue damage in the foot, often accompanied by neuropathy and/or peripheral artery disease in the lower extremities of individuals with diabetes (1). DFU typically manifests as a full-thickness wound in the dermis, usually found in weight-bearing or exposed areas below the ankle. The global prevalence of diabetic foot ulcers (DFUs) is estimated to be 6.3%, with a higher incidence observed in individuals with type 2 diabetes than in those with type 1 diabetes. DFUs were primarily managed by nurses in the community, with only 5% of patients seeking assistance from a podiatrist or receiving a pressure offloading device. Among the cases, 35% of DFUs healed within 12 months, 48% remained unhealed, and 17% of wounds necessitated amputation during the same period. The average National Health Service (NHS) expenditure on wound care over 12 months was estimated at £7800 per DFU, with costs ranging from £2140 to £8800 per healed and unhealed DFU, respectively. In addition, the cost increased to £16,900 per amputated wound (2). The article published (3) by American Diabetes Association (ADA) highlights the life style management in diabetic patient which includes Diabetes self-management education and support (DSMES), nutrition therapy, physical activity and psychosocial care.

**Figure 1:**
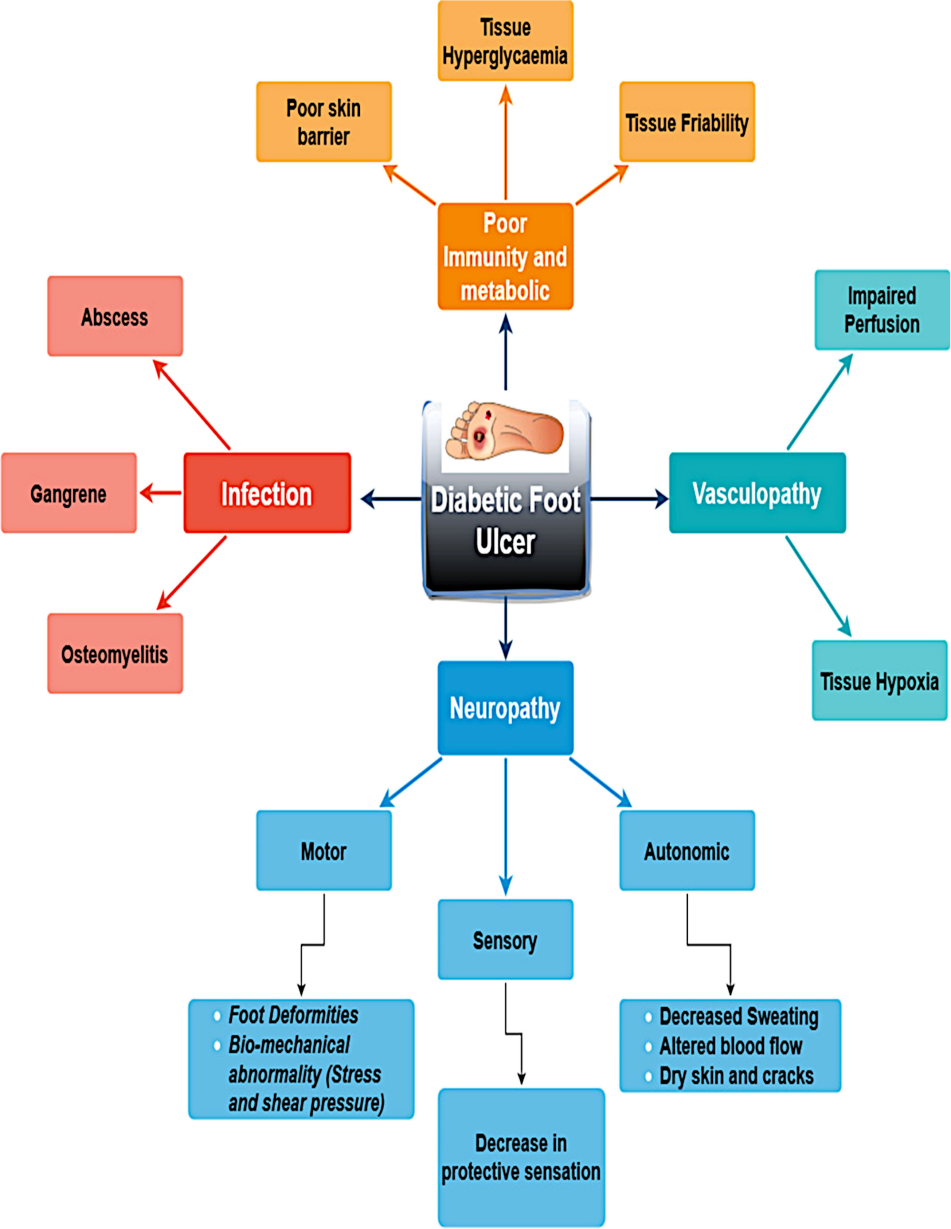
Pathophysiology of diabetic foot ulceration.

DFUs evolve from intricate interactions of multiple pathophysiological mechanisms linked to diabetes mellitus. The main causes of diabetic foot ulcers are infections caused by vasculopathy, poor metabolism or immunity, and neuropathy. A few significant risk factors for the development of DFU are shown in **Figure 1**. Effective healing is hindered by peripheral arterial disease, which decreases blood flow. Hyper-glycemia weakens the immune system and makes people more vulnerable to infection. Pressure points and ulcer development are facilitated by abnormalities in foot biomechanics. Microvascular damage and chronic inflammation further hinder normal wound healing in patients with diabetes.

Lower extremity amputation is a severe consequence of diabetic foot complications, with ulcers preceding amputation in approximately 85% of cases. The presence of diabetic foot ulcers significantly increases mortality rates among patients with diabetes, surpassing that of diabetes alone (4). This elevated mortality risk persists even after adjusting for chronic kidney and cardiovascular diseases (5). Although the precise mechanism underlying this excess mortality remains unclear, it is likely that the presence of foot ulcers in diabetes interacts with other cardiovascular risk factors or is associated with potential inflammation (6). Brownrigg et al. (6) conducted a statistical analysis of 3619 deaths, revealing that individuals with diabetes have a 1.89 times higher risk of mortality when they also have diabetic foot ulcers compared to those without foot complications (95% CI, 1.60–2.23). Similarly, Saluja et al. (7) reported findings regarding mortality rates. A survey conducted on diabetic foot ulcers in North India identified risk factors such as older age (>50 years), longer duration of diabetes (4–8 years), tobacco use, insulin administration, and rural residence patients (8).

In a recent study, Zhang et al. (9) investigated the global impact of diabetic foot ulcers (DFUs) and gathered data from various sources, including PubMed, EMBASE, ISI, and Cochrane. The findings revealed that males were more likely to experience foot complications than females, with DFUs being more severe in patients with a low body mass index, prolonged diabetes, hypertension, and a history of smoking.

Notwithstanding well-established guidelines, DFU treatment has not yielded satisfactory clinical outcomes. To address this complex disease, a precise clinical assessment considering the patient’s clinical context and wound condition should be conducted, adhering to an evidence-based treatment protocol. Presently, DFU clinical procedure includes bed rest, administration of antiseptic cream, and a prolonged surgical procedure. However, these conventional procedures lack continuous monitoring prediction of wound healing progress, which may lead to complications in diabetic patients. Thus, interdisciplinary modern technology for real-time screening and accessibility to diabetes education can effectively support treatment procedure and prevention.

The Fortune Business Insights report published in September 2024 indicated that the global diabetic foot ulcer treatment market size was valued at USD 8.33 billion in 2023. The market is projected to grow from USD 8.83 billion in 2024 to USD 14.37 billion by 2032 (10). This report highlights a transition from traditional wound treatment procedures towards advanced wound care methodologies in terms of dressing techniques (such as hydrocolloids, hydrogel, and foam dressings engineered to control moisture) and the development of new compact devices [such as the RENASYS EDGE NPWT system (11)] for home-based wound care.

The rising prevalence of diabetes poses significant medical and economic challenges worldwide. As a result, preventive measures, such as annual diabetic foot screening and multidisciplinary diabetic foot care, supported by portable, low-cost advanced equipment, have been implemented to identify high-risk diabetic patients early.

There are several studies that have evaluated the costs related DFU management (12, 13). These costs analysis is summarized in **Table 1** below:

**Table 1:** Cost associated with diabetic foot disorders.

Country	Cost USD	Type of care
France	1265 annually	DFU
UK	7539/patient	DFU
Sweden	24965/patient	without amputation
	47518/patient	with minor amputation
	42858/patient	with major amputation
India	1960 annually	DFU
Brazil	306 annually	DFU
Saudi Arabia	1783 annually	DFU
Nigeria	1104 annually	DFU
US	10.9 billion annually	Foot care

It is crucial to provide education about diabetes and diabetic foot care to literate laypeople, utilizing clear and understandable language to eliminate the stigma surrounding diabetic foot ulcers. Employing a structured approach can offer language-based information that empowers individuals with diabetes to take charge of their health and prioritize preventive care. Encouraging regular self-monitoring and prompt action in the event of any issues can greatly reduce the risk of diabetic foot complications. Furthermore, technological advancements in diabetic foot care accompany traditional methods by providing personalized, effective, and prompt interventions that improve outcomes for patients with diabetic foot ulcers (DFUs). Incorporating these technologies into clinical practice enhances the overall management of diabetic foot complications, reduces the risk of complications, and improves quality of life. These advanced technologies not only facilitate early detection and diagnosis of DFUs but also empower both patients and healthcare providers to take proactive steps in managing diabetic foot health effectively. Thus, it is crucial to conduct comprehensive research to for the advancement in technology to address the complications associated with DFUs. Markakis et al. (14) highlighted the need for standards and guidelines to conduct trials for potential diabetic foot treatments.

## 2 Severity classification

In this section of the text, the authors aim to emphasize the severity level and its characteristics in order to delineate the management of DFUs The purpose of this discussion is to provide a comprehensive understanding of the DFU management framework. Diabetic foot ulceration is more prevalent in men than in women and in patients with type 2 diabetes mellitus compared to those with type 1 diabetes mellitus. It is crucial to classify diabetic foot ulceration to determine the severity of the condition and implement appropriate management strategies.

Various researchers have proposed classification and scoring systems to grade the risk levels in DFU. For instance, the Bates Jensen Wound Assessment Tool (BWAT) consists of 13 score items, with each item level from to 0-5 (15). Similarly, Chetpet et al. (16) used 13 score items, with variable points designated for each variable. In 2002, Margolis et al. (17) proposed 6 grade system similar to the Wagner scale based on depth, infection, and gangrene, and is known as the Curative Health Services (CHS) score. The other listed scoring scales are DEPA (depth, extent of bacterial colonization, phase of healing, and associated etiology), CSSC (Clinical signs and symptoms checklist), DFU (Diabetic foot infection), DFUAS Diabetic foot ulcer assessment scale, and DIAFORA Diabetic foot risk assessment etc. Thus, various classification and scoring systems are well summarized and compared by researchers (18). The most frequently adopted assessment systems are (Meggitt-) Wagner, UTWS (University of Texas Wound classification system) (19), WIFI, SINBAD (20) (site, ischemia, neuropathy, bacterial infection, Area, Depth), PEDIS (perfusion (PAD), extent (area), depth, infection, and sensation (neuropathy) (21), S(AD)SAD (size (area and depth), sepsis, arteriopathy, and denervation (22).

PEDIS is graded as high-versus low-risk in DFU. Numerous researchers have adopted this scoring system and concluded that the PEDIS score helps to predict lower extremity amputation (major) and morality, but not healing (22). Refined from the S(AD)SAD, the SINDBAD score system predicts DFU clinical outcome (healing and LEA) and cost. The SINBAD system still contains five elements (area, depth, infection, ischemia, and neuropathy) and grades each element as either 0 or 1 point to create an evaluation system with scores of 0-6 to description of increasing severity (23). The University of Texas proposed the UTWCS score system based on DFU symptoms to predict healing time, LEA, and cost. This system is more helpful in predicting amputation than the Meggitt-Wagner system (24).

The system was initially proposed by Meggitt in 1976 and subsequently disseminated by Wagner in 1979, however, it does not account for clinical parameters such as peripheral neuropathy and PAD. As a result, it cannot differentiate between infection and ischemic lesions, which is also a factor in its recognized lack of precision and limitations (25). The severity of DFUs can be systematically assessed using the Wagner system, which uses a scale ranging from 1 to 5 to categorize ulcer severity based on the depth of the ulcer and the extent of tissue involvement, as shown in **Table 2**.

**Table 2:** Wagner’s classification of diabetic foot ulcers (1).

Grade	Characteristic
Wagner grade 1	Partial- or full-thickness ulcer (superficial)
Wagner grade 2	Deep ulcer extending to ligament, tendon, joint capsule, bone, or deep fascia without abscess or OM
Wagner grade 3	Deep abscess, OM, or joint sepsis
Wagner grade 4	Partial-foot gangrene
Wagner grade 5	Extensive gangrene involving the entire foot.

In 2014, the Society for Vascular Surgery Lower Extremity Guidelines Committee proposed the Wound, Ischemia, and foot Infection (WIfI) system, which identified three critical risk factors that can lead to the amputation of lower limbs. The foot infection classification system, known as WIfI, was developed to combine all three variables [Wound (W), Ischemia (I), and Foot Infection (fI)] to assess the risk of limb loss in patients with diabetic foot ulcers. Armstrong et al. (26). summarized these 3 variables on a scale of 0-3 according to the severity. A higher WIFI score is associated with amputation and morbidity and can be used to determine the need for revascularization. WIfI scores of 1, 2, 3, and 4 were associated with 1-year amputation rates of 0, 8, 11, and 38%, respectively. Mills et al. (27) presented the amputation risk and clinical management based on the WIFI score. They presented the summarized WIfI score to identify amputation risk (after 1 year, Pl. ref. **Table 3**) and information for determining whether the patient will require revascularization (Pl. ref. **Table 4**). These classification systems assist a structured approach for healthcare professionals to evaluate, diagnose, and manage foot infections by considering various factors influencing treatment decisions and outcomes.

**Table 3:** Estimate risk of amputation at 1 year (27).

	Ischemia-0				Ischemia-1				Ischemia-2				Ischemia-3			
**W-0**	VL	VL	L	M	VL	L	M	H	L	L	M	H	L	M	M	H
**W-1**	VL	VL	L	M	VL	L	M	H	L	M	H	H	M	M	H	H
**W-2**	L	L	M	H	M	M	H	H	M	H	H	H	H	H	H	H
**W-3**	M	M	H	H	H	H	H	H	H	H	H	H	H	H	H	H
** **	**fI-0**	**fI-1**	**fI-2**	**fI-3**	**fI-0**	**fI-1**	**fI-2**	**fI-3**	**fI-0**	**fI-1**	**fI-2**	**fI-3**	**fI-0**	**fI-1**	**fI-2**	**fI-3**

W, Wound; I, Ischemia; fI, foot Infection.

Green, Very low risk; Yellow, Low risk; Burnt Orange, Moderate Risk; Red, High Risk.

**Table 4:** Estimate of benefit of/requirement for revascularization (27).

	Ischemia-0				Ischemia-1				Ischemia-2				Ischemia-3			
**W-0**	VL	VL	VL	VL	VL	L	L	M	L	L	M	M	M	H	H	H
**W-1**	VL	VL	VL	VL	L	M	M	M	M	H	H	H	H	H	H	H
**W-2**	VL	VL	VL	VL	M	M	H	H	H	H	H	H	H	H	H	H
**W-3**	VL	VL	VL	VL	M	M	M	H	H	H	H	H	H	H	H	H
** **	**fI-0**	**fI-1**	**fI-2**	**fI-3**	**fI-0**	**fI-1**	**fI-2**	**fI-3**	**fI-0**	**fI-1**	**fI-2**	**fI-3**	**fI-0**	**fI-1**	**fI-2**	**fI-3**

W, Wound; I, Ischemia; fI, foot Infection.

Green, Very low risk; Yellow, Low risk; Burnt Orange, Moderate Risk; Red, High Risk.

## 3 Management of diabetic foot ulceration

The management of diabetic ulcers involves determining and improving the underlying cause, wound care, and prevention of ulcer recurrence through debridement, offloading, managing infection, and using clean and moist wound dressings. The main goal of the management of diabetic ulcers is wound closure. This is evidence for the use of home monitoring of foot skin temperatures and therapeutic footwear to prevent recurrent foot ulcers. Additionally, some evidence suggests that integrated foot care is effective in preventing recurrent foot ulcers (1). Comprehensive glycemic management is essential for accelerating the healing process. To increase the blood flow for healing, vascular evaluation and revascularization procedures might be considered. Modern solutions for the revascularization of peripheral foot arteries in cases of foot ischemia include endovascular techniques such as angioplasty, stenting, and sub intimal recanalization (28). These techniques have been found to be feasible and safe, with good success rates for lower-limb preservation (29). The use of new interventional and vascular surgical procedures, particularly in the arteries of the leg and foot, is effective in preventing major amputations (30). The shift from bypass surgery to less invasive endovascular procedures as the first-choice revascularization technique has resulted in a significant change in the treatment of critical limb ischemia (31).

A range of revascularization techniques has been explored for the treatment of foot ischemia. Sacheck (32) and Shapovalov (33) both emphasized the importance of individualized approaches, with Shapovalov (33) specifically highlighting the effectiveness of differentiated revascularization techniques in achieving amputation-free survival, wound healing, and foot support function. Angiosome-targeted revascularization, as discussed by Biancari (34), has shown promising results in terms of improving wound healing and limb salvage rates, particularly when feasible. These studies collectively underscore the need for further research to establish clear guidelines for the selection and application of revascularization methods for foot ischemia. Hendri et al. (35) conducted a Chi-Squared comparative analysis on 23 patients, revealing that the wound healing rates for re-vascularized patients (i.e., 78.3%) were higher than those for non-vascularized patients (26.1%). Additionally, revascularization is a necessary intervention for patients with ischemic foot ulcers to prevent major amputations, as indicated by Meloni et al. (36). Moxey and Chong (37) have advocated that stem cell therapy is a promising area of research for treating ischemia in DFU, particularly when revascularization treatment is not possible. Meaningful outcomes of treatment depend on classifying patients according to the severity of their arterial disease anatomy and the degree of tissue loss.

The prevention of complications and efficient healing of diabetic foot ulcers are contingent on regular monitoring, patient education, and multidisciplinary care. In severe cases, surgical intervention, such as amputation, may be necessary. These techniques are classified in **Table 5** as non-invasive and invasive modalities for a clear overview.

**Table 5:** Overview of diabetic foot management strategies (38).

Treatment modality	Level of evidence	Strength of recommendation
*Non-invasive modalities*		
Would dressing	High	Strong recommendation
Antibiotics	Low to moderate	
Total-contact casting and pressure offloading techniques	High	Strong recommendation
Maggot therapy	Low	Weak recommendation
Hyperbaric oxygen	Low	Weak recommendation
Topical growth factors	Moderate	Could be beneficial
Shock wave therapy	Low	Could be beneficial
Cell therapy	Low	Weak recommendation
*Invasive modalities*		
Debridement	Moderate to high	Strong recommendation
Skin grafting	Moderate	Could be beneficial
Revascularization	Moderate	Strong recommendation

The strategies and probabilities of Diabetic Foot Ulcer (DFU) occurrence and follow-up frequency have been outlined by researchers (39, 40). These follow-up measures and their characteristics aid practitioners in assessing risk levels and devising recommendations for subsequent medical treatment. **Table 6** presents an effective risk assessment strategy that can be employed to evaluate a patient’s likelihood of developing diabetic foot complications.

**Table 6:** Shows the DFU risk level strategy and its Follow-up (41, 42).

Risk Level	Risk Factors	Characteristics	Follow-Up frequency
Low Risk	No risk factors present	No loss of protective sensation or peripheral artery disease	Annually
	Presence of callus formation alone	Loss of protective sensation or peripheral artery disease	6-12 months
Moderate Risk	Deformity or Previous ulceration	≥2 Factors among loss of protective sensation, peripheral artery disease, and foot deformity	3-6 months
	Non-critical limb ischemia		
High risk	Previous ulceration	In remission: history of diabetic foot ulcer, amputation (minor or major), or end-stage renal disease	1-3 months
	Previous amputation		
	On renal replacement therapy		
	Neuropathy and non-critical limb ischemia	Active ulcer, Charcot arthropathy, or infection with or without peripheral artery disease	Rapid referral to specialist/ multidisciplinary team
	Neuropathy with callus and/or deformity		
	Non-critical limb ischemia with callus and/or deformity		

## 4 Technology assisted approach

Advanced technologies have a significant impact on the early detection and diagnosis of Diabetic Foot Ulcers (DFUs). They offer several advancements that improve patient outcomes, such as temperature monitoring devices, pressure sensors, imaging techniques, wearable devices, telemedicine, and AI-assisted treatment. Additionally, advanced technology provides innovative solutions that promote healing, including artificial skin, advanced wound healing, negative-pressure wound therapy, and laser and electrical therapy. The aforementioned technologies (such as laser Doppler flowmetry, Doppler ultrasound, plantar pressure and pressure gradient system, and ultrasound indentation tests) for diabetic foot assessment can be conducted within a 2-hour timeframe. It is recommended that an integrated care team, comprising health technology or biomedical engineering professionals, be incorporated to perform these assessments routinely, thereby establishing a baseline for each individual with diabetes (43). Lung et al. (43) presented the emerging modern techniques for the assessment of DFU. These include sensory testing with monofilaments for neuropathy tests, Doppler ultrasonography, or laser Doppler flowmetry for peripheral arterial disease. With the advent of AI systems in smartphone applications, cloud-based technologies can facilitate remote screening for the detection of DFUs (44).

In the following sections, the authors will delve into some of these advanced technologies.

### 4.1 Plantar pressure distribution management

Diabetic foot issues are a global concern, as they result in significant social, medical, and financial difficulties for both patients and their families. Foot ulcers are the most common diabetes-related complication, and they are more likely to cause neuropathic pain than any other issue. To prevent the recurrence of diabetic foot ulcers (DFUs) or neuropathic damage, healthcare professionals traditionally utilize a manual method of cutting insoles at the site of DFUs. However, this approach is time-consuming and may not be as accurate as desired. To address this challenge, researchers have conducted various studies to develop customized insoles for diabetes patients. In the context of customized insole development, Nesali et al. (45) utilized an open computer vision (CV) library and pixel-distribution analysis to create accurate customized insoles for individuals with DFUs. The team also introduced the “Diabisol” web-based application, which can detect the wound area and size, identify high-pressure areas on the foot, and delineate sites requiring pressure offloading.

**Figure 2:**
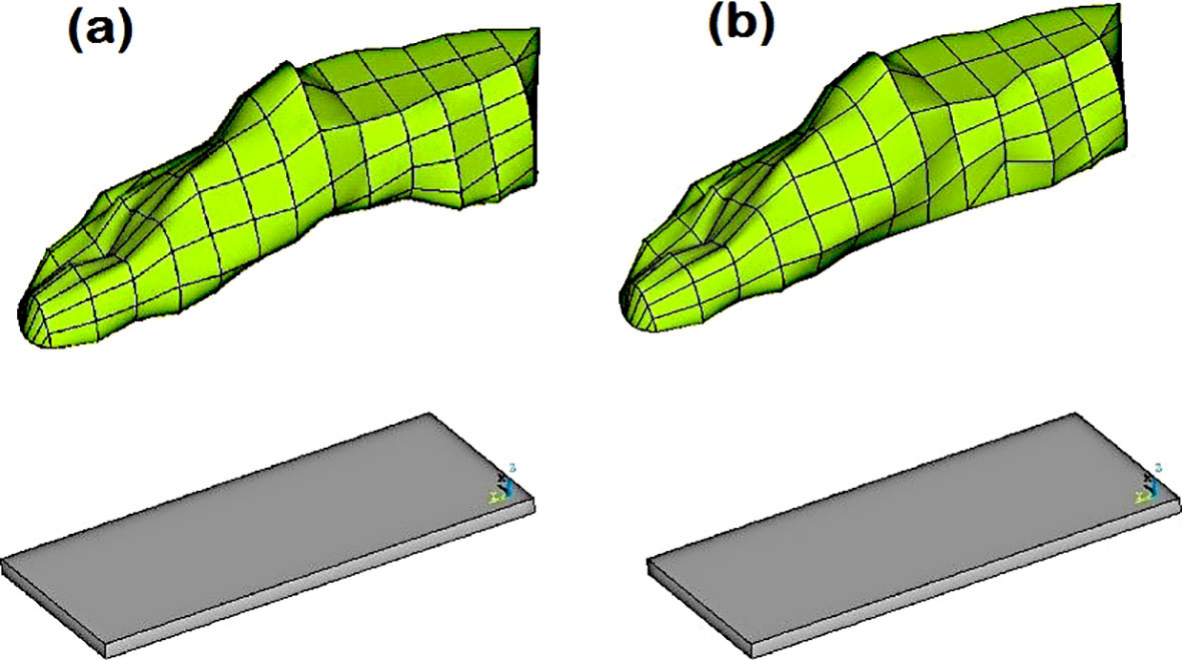
**(A)** Normal foot model with the custom ground, and **(B)** Flat foot model with the custom ground (41).

**Figure 3:**
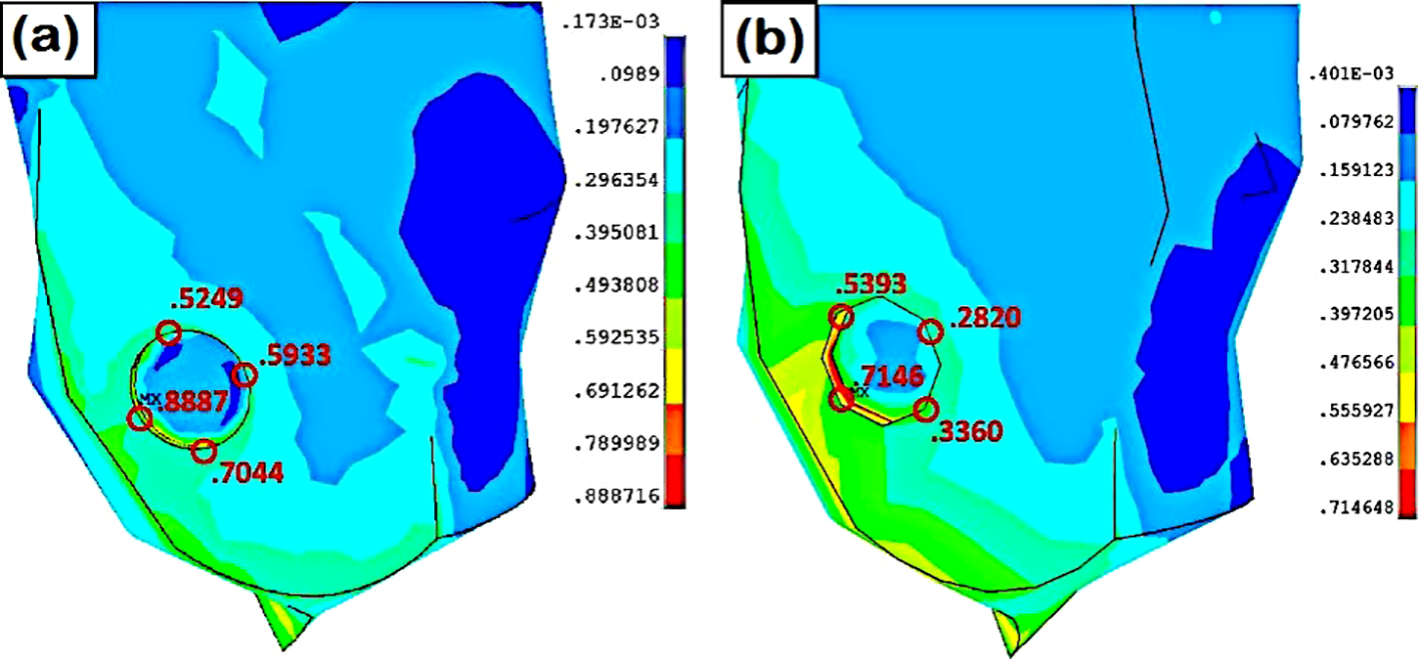
Stress at the lateral heel ulcer **(A)** normal foot, **(B)** flat foot (41).

There are studies on the prevention and treatment of diabetic foot, but only limited studies have reported the biomechanics of diabetic foot ulceration and its further progression. Using measurements obtained from a human subject, Singh et al. (41) created a full-scale foot model. They used computational modeling to simulate ulcers of various sizes and depths at several plantar regions. In order to investigate the impact of flat foot circumstances on identical diabetic ulcers, the foot model was also computationally modified (see **Figure 2**). A standing position was considered, and the stress produced by the plantar region was examined. The highest stresses in the heel area were recorded (refer to **Figure 3**), demonstrating the danger of ulcer recurrence. A significant study was conducted by Gupta et al. (42) and investigated the impact of all potential ulcer sites on the plantar peak stresses created and the peak stress locations where new ulcers might develop. Using a full-scale foot model, 52 ulcer sites were independently simulated under both walking and standing loads. In order to create unique formulations for forecasting peak plantar stresses and their positions for any given ulcer site, the produced stresses were normalized with the size of the foot and statistically evaluated. Such studies are expected to be crucial for the development of appropriate therapies, such as medical interventions (e.g., insoles and customized orthotics) for the treatment of diabetic foot ulcers (46, 47).

The administration of DFUs is aided by the introduction of modern computational tools, which have yielded good results.

**Figure 4:**
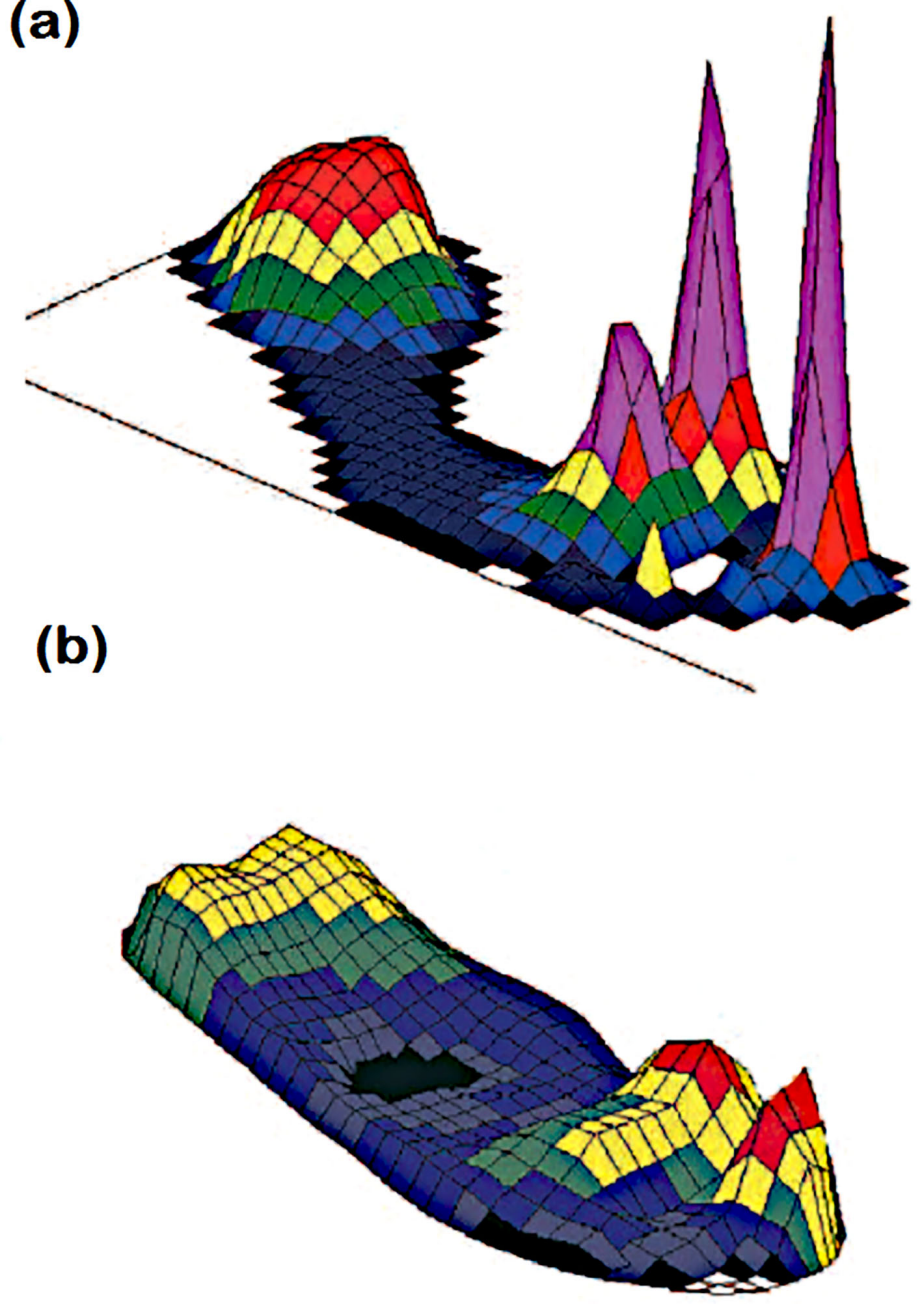
Plantar pressure distribution under the foot during **(A)** bare foot walking and **(B)** walking in appropriate therapeutic shoes and custominsoles181Peak pressures are more than 1000 kPa in **(A)** and less than 200 kPa in **(B)**. This patient had previous ulcers at the site of raised pressure under the hallux (47).

According to a related study on diabetic foot healing (48), pressure ulcer alleviation is always advised and should be considered when treating the condition. Deformation of the bones might cause significant plantar pressure and slow down the healing process. As a result, pressure management between the foot and the shoe as well as between the foot and the ground relieves strain and accelerates the healing process. For barefoot walking, **Figure 4** illustrates the distribution of plantar pressure under the foot, with a focus on bespoke insoles and therapeutic shoes.

### 4.2 Computer-assisted mapping and prediction

Globally, approximately 15%–25% of diabetic people suffer from diabetic foot ulcers. The traditional methods employed for DFU diagnosis are susceptible to human error and necessitate a high level of expertise and experience. The utilization of computer-assisted diagnosis, which is widely accepted in the manufacturing sector, is also gaining traction in the medical sector. This approach not only reduces costs but also enhances accuracy. In the medical field, diabetic foot ulcers have been diagnosed using various sensor technologies for decades to detect or regulate harmful foot pressure in patients with diabetes. The emergence of DFUs in patients with diabetes can be attributed to two factors: causative factors and contributing factors. Sensory neuropathy, which causes loss of sensation and increases the risk of foot ulcers (8), is the primary causative factor. Another causative factor is motor and autonomic neuropathy, which leads to abnormal bone growth and skin dryness, resulting in high plantar pressure and the development of foot ulcers. Contributing factors, such as peripheral vascular disease (atherosclerosis), collagen cross-linking disorder, and immunological disorders in diabetic patients, can delay wound healing (49).

However, in modern digital healthcare systems, medical imaging coupled with machine learning and deep learning has been widely applied in various diseases, such as cancer detection and customization of medicine for various diseases. Puneeth et al. (50) proposed EfficientNet neural network model for early detection of and prognosis of DFU. They utilized 844-foot images of healthy and diabetic ulcerated feet for the precise identification of ulcers.

In modern medical industries, medical image databases assisted by Artificial Intelligence (AI) are the best source of information about patients to address the challenges related to the management of treatment and shortage of skilled staff. The introduction of AI in medical imaging databases enhances the accuracy and efficiency of diagnoses. This computational AI-assisted analysis accurately diagnoses patient conditions and is widely used in engineering applications (51) as well as for environmental issues (52). Toofanee et al. (53) adopted an artificial intelligence (AI) tool and deep learning for DFU classification and detection. They proposed the integration of EfficientNet (Convolutional Neural Network) and Vision Image Transformers within the Siamese Neural Network architecture for the categorization of DFU images into four different classes: none, infection, ischemia, or both. This classification aids medical practitioners in managing the treatment of patients with DFU. The use of this cutting-edge diagnostic strategy has encouraged researchers to combine computational methods with the medical field in order to treat complex DFUs. A thorough analysis of the methods in which different researchers have applied artificial intelligence (AI) to support DFU monitoring methods was presented by Maria et al. (54) They also discussed the advantages of these techniques and the challenges of applying them to remote patient care. This is because of the high cost involved and the portability of the equipment. Many factors influence the monitoring of DFU healing, and measuring these factors requires the use of several expensive devices. Thus, the availability of a database may reduce expenses and allow DFU patients to receive timely and precise medications to alleviate this limitation. In addition to the machine learning approach, Cassidy et al. (44) presented a study on automated DFU detection using a smartphone and cloud-based architecture. They also demonstrated a high sensitivity (0.9243) of the system for the detection of DFU.

**Figure 5:**
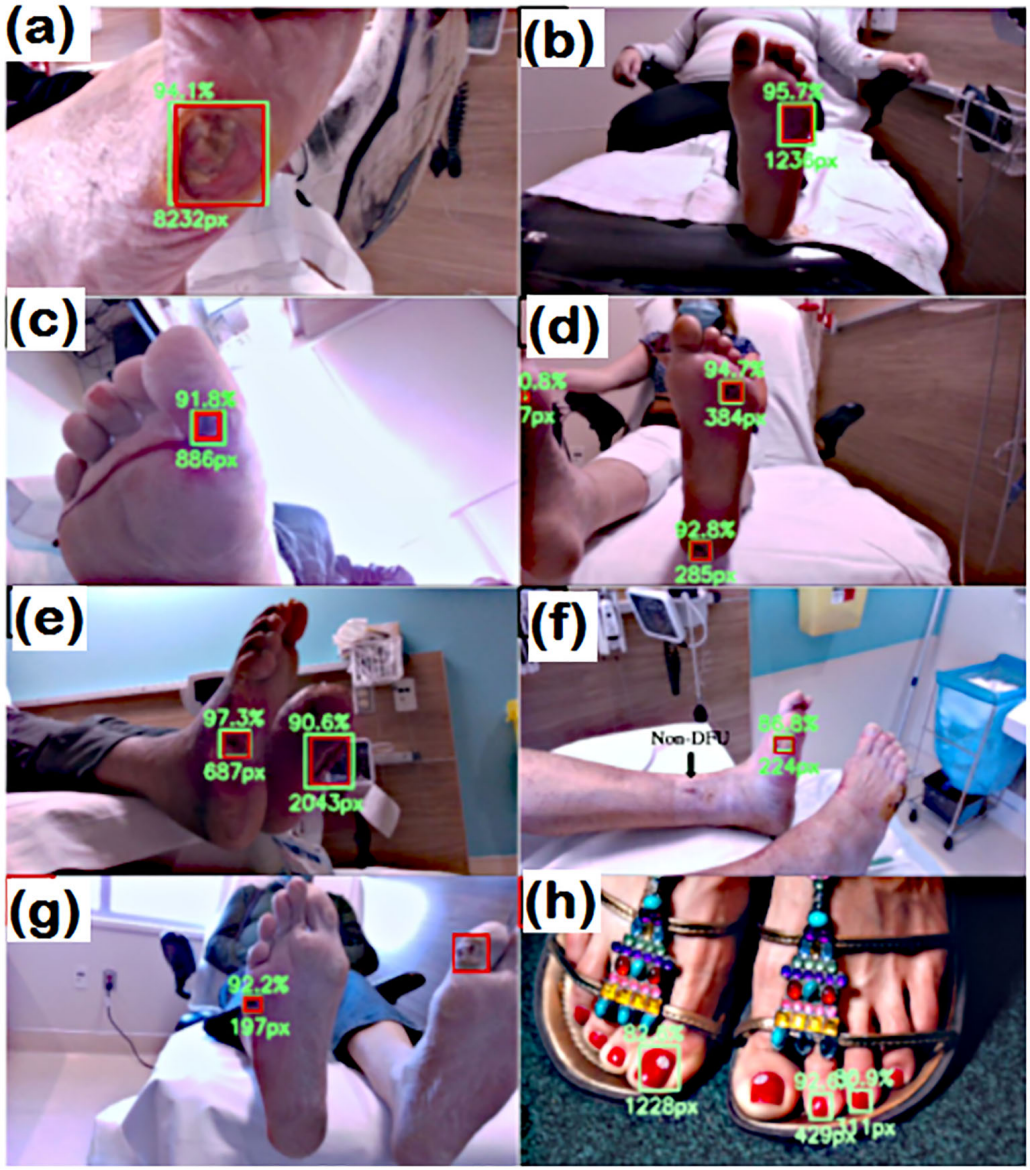
Detection capabilities of trained network. RED: Actual location GREEN box **(A–H)**, however, the predicted location of wound. In **(G)** the wound detection fails. and **(H)** false prediction due to nail polish (55).

In addition to the machine learning approach, Cassidy et al. (44) presented a study on automated DFU detection using a smartphone and cloud-based architecture. They also demonstrated a high sensitivity (0.9243) of the system for the detection of DFU. The sensitivity of DFU detection using an AI-based computational approach was thoroughly demonstrated by Reza et al. (55) They established and tested a deep-learning network employing EfficientNet and UNet architectures for DFU prediction. The network was trained by collecting datasets from 269 patients with DFUs and 3700 RGB and thermal images. **Figure 5** shows the results of the trained network for predicting the DFUs.

### 4.3 Internet-based self-management and monitoring

The emerging technology evident that internet based self-management service is beneficial for the management of type 2 patients (56, 57). However, it will be more effective when the two-way communication between patient and health care team (58). Lazarus et al. (59) examined the most recent advancements and practical uses of digital health technologies.

## 5 Traditional and innovative approaches for the treatment of DFUs

**Figure 6:**
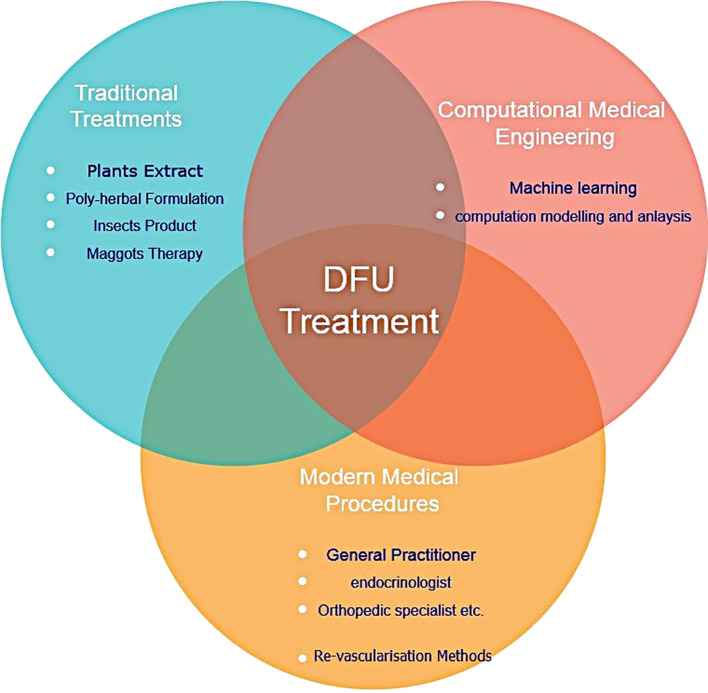
Various DFU treatment methods.

It is crucial to remember that even though the traditional approach is still valid, advancements in technology and therapeutic modalities have increased the opportunities for treating DFUs and improving patient outcomes. Based on National Institute of Health and Clinical Excellence strategies, a multidisciplinary team consisting general medicine, a nurse, orthotic specialist, vascular surgeon infection disease specialist, dietician can manage DFU more efficiently (60). However, author advise to integrate the specialist from engineering background with medical (Modern and Traditional) specialist for more effective treatment of DFU. **Figure 6** represents graphic illustration of several DFU methods of treatment

### 5.1 Traditional medicine approach

The traditional medicine approach refers to medical practices, therapies, and treatments that have been developed over centuries in various cultures around the world. These practices often stem from indigenous knowledge, passed down through generations, and deeply rooted in cultural beliefs, local resources, and societal norms. The potential of traditional medicine prepared from herbs, encompasses a wide range of practices such as acupuncture, Ayurveda, traditional Chinese medicine or larval debridement therapy has been highlighted by researchers. Despite its long history and cultural significance, traditional medicine faces challenges in terms of standardization, regulation, and integration with modern healthcare systems. Efforts are being made to bridge the gap between traditional and modern medicine, recognizing the potential benefits of combining both approaches to improve overall health outcomes. Some traditional procedures are also granted patents (pl. refer **Table 7**). Abhijit et al. (62) examined DFU management strategies based on traditional medicine. They advocated that a traditional approach (such as larval debridement therapy or treatment with naturally occurring acids) integrated with modern medicine would benefit the patent with DFU.

**Table 7:** Diabetics foot ulcer occurrence model (61).

Parameters	Hazard ratio (α = 0.05)	P-Value
AIC	1.10 (1.06-1.15)	Most significant factor (p<0.001)
impaired Vision	1.48 (1.00–2.18)	Significant factor (p<0.05)
Foot ulcer History	2.18 (1.61–2.95)	Most significant factor(p<0.001)
Amputation History	2.57 (1.60–4.12)	Most significant factor(p<0.001)
Monofilament insensitivity	2.03 (1.50–2.76)	Most significant factor(p<0.001)
Onychomycosis	0.73 (0.54–0.98)	Significant factor(p<0.035)
Tinea pedis	1.58 (1.16–2.16)	Significant factor (p<0.004)

### 5.2 Innovative approaches

Innovative strategies for managing diabetic foot ulcers (DFUs) aim to increase healing rates, lower infection risks, avoid amputation, and improve the general quality of life of patients with diabetes. Bioengineered skin substitutes, growth factors, extracellular matrices, antimicrobial dressings, hyperbaric oxygen therapy, biofilms—communities of bacteria encased in a protective extracellular matrix—are a few examples of these inventive techniques.

DFU causes peripheral artery disease or neuropathy, which creates a favorable environment for bacterial growth, resulting in chronic infections. This chronic infection may be treated with medication or surgery; however, if it fails, the only option is amputation. Marta et al. (63) proposed organic light-emitting diodes with low irradiance, based on photodynamic therapy, to inactivate a high percentage of antibiotic –resistant clinical strains.

Clinical guidelines often state that DFU wound sampling and microbiological analysis are generally performed only if infection is suspected. However, it is reported that subclinical infections, biofilm encased bacterial hamper the wound healing process. Armstrong et al. (26) therefore proposed using fluorescence imaging to detect bacterial lance in wounds, allowing for effective treatment monitoring and early infection control. According to Li et al. (64), a poly(Penta-hydro-pyrimidine) library-synthesized hybrid hydrogel significantly increased the rate of healing of infected DFU, achieving a 92% healing rate in just 10 days. The hydrogel was made in one pot method (65) using the amine (-NH2) and phenyl boric acid (-B(OH)2) additions, which improved the hydrogel’s biocompatibility and antibacterial activity.

In recent years, the significant increase in medical nanomaterials has substantially contributed to tissue regeneration. Hydrosol-assisted nanomaterials possess physicochemical and mechanical properties that effectively manage wounds according to repair requirements and provide support for tissue regeneration (66). Singh et al. addressed the challenges of designing nanomaterials for wound healing and ulcer management, including their biomechanical properties and capacity to inhibit bacterial growth (66). In the field of nanomedical materials, the wound healing properties of silver nanoparticles have also been evaluated (67). The antibacterial properties of carrageenan silver nanoparticle composite acticaot demonstrate efficacy in treating diabetic wound infections, characterized by dense deposition of collagen.

By leveraging these innovative procedures, healthcare providers can optimize DFU management, improve clinical outcomes, and enhance the quality of life of individuals with diabetes. Furthermore, the field of wound care is continuing to advance because of ongoing research and technology advancements, which gives optimism for future advancements in the management of DFU. The authors provide some examples of statistical techniques for predicting the most effective way to manage DFU medication in the following section.

### 5.3 Statistical approach

In addition to computational machine learning methodology, Boyko et al. (61) conducted a study on the occurrence of DFU using statistical analysis of the clinical data collected for an average patient age of 62.4 ± 10.8 years. They proposed model for DFU prediction utilized backward step algorithm and final Cox regression model is as follows: (**Table 7**)

The study results showed that after 1 year and 5-years of follow-up, the final multivariable model’s prediction accuracy was assessed using the area under the receiver operating characteristic (ROC) curve, which showed good accuracy or development of DFU. In another statistical analysis (68), the correlation between diabetic retinopathy and DFU was investigated. The analysis revealed no significant association between these complications (P = 0.744). However, the presence of diabetic retinopathy, high HbA1c, high serum creatinine, old age, high pulse pressure, low cholesterol, and low BMI were contributing factors to DFU complications. Thus, the researcher advocated for retinal examination of patients with DFU, especially those with higher serum creativity. From this study, it can be concluded that long-term uncontrolled glucose levels will lead to complications and DFU is one of those associated with other alignments.

The statistical computation approach was also adopted by Imam et al. (69) to measure the outcome of the effect of hyperbaric oxygen therapy for the treatment of DFU. During this process, the patient was kept in a chamber with 100% oxygen at an atmospheric pressure higher than sea level. The study was conducted on 7219 patients with DFU, and the results showed that hyperbaric oxygen significantly enhanced ulcer healing and lower mortality compared to the standard treatment given to patients with DFU.

## 6 Critical aspects and limitations in emerging technologies

In the comparison of traditional and emerging technologies for diabetic foot ulcer management, several critical aspects come into play, such as, diagnosis, treatment, monitoring, and patient engagement (ref **Table 8**).

**Table 8:** Comparison of key aspect of traditional methods and emerging technologies.

Critical aspects	Traditional method	Emerging Technologies
Diagnosis	a. Visual Inspection • Wound size and depth assessment • Tissue color and texture evaluation. b. Wound Assessment • Probe-to-bone test • Monofilament testing for neuropathy	a. Smart Imaging Techniques • Smart image technologies • Hyperspectral imaging • Thermal imaging b. AI and Machine Learning Algorithms • Automated wound classification systems • Predictive model for wound healing • Risk stratification algorithms
Treatment	a. Debridement • Sharp debridement • Enzymatic debridement • Autolytic Debridement b. Dressings • Gauze dressing • Hydrocolloid dressing • Foam dressing c. Offloading • Contact casting • Removable cast walker • Therapeutic foot ware	a. Advanced Dressings • Smart dressing with embedded sensors • Nanofiber dressing • Growth factor releasing dressing b. Telemedicine • Remote wound assessment platforms • Virtual consultations with specialist • Tele-monitoring wound progression c. Biological therapies • Gene therapy approach • Bioengineered skin • Stem cell therapy
Monitoring	a. Regular Check-ups • Frequency of follow-up visits • Physical examination procedure b. Manual Documentation • Paper based wound documentations • Patient reported outcomes.	a. Wearable Devices • Smart insole for pressure monitoring • Continuous glucose monitoring systems b. Mobile Apps • Wound track app • Medication reminder systems
Patient Education	Printed Materials • Broachers on foot care • Dietary guidelines • Medication adherence instructions	a. Virtual Reality (VR) and Gamification • Interactive educational programs • Simulated wound care scenarios • Motivational games for adherence b. Remote Patient Monitoring • Home based wound imaging devices • Automated alert systems for complications • Real time data sharing with specialist

### 6.1 Advantages and limitations of emerging technologies

These advanced technologies facilitate more accurate diagnostics and treatments tailored to individual patient requirements with precise responses to monitor wound status. This real-time monitoring and feedback minimizes complications and reduces the economic burden. Patients and healthcare providers can work together more effectively, leading to improved management of diabetic foot ulcers. These technologies can also contribute to enhancing patient education and adherence through interactive tools. The incorporation of emerging technologies into clinical guidelines substantially reduces DFU complications. By utilizing interactive tools, data-driven insights, and real-time feedback, both patients and healthcare providers can collaborate more effectively, resulting in improved management of diabetic foot ulcers. The adoption of emerging technologies in DFU management faces several limitations, barriers, and regulatory challenges. These advanced technologies may entail higher initial costs and necessitate investment in training and infrastructure, which presents a significant challenge for underdeveloped countries. The healthcare team requires extensive training and incurs maintenance costs to effectively utilize new technologies. The continuous evolution of technology also required ongoing education and infrastructure to support advanced technologies. Moreover, obtaining regulatory clinical approval for new technologies (e.g., FDA in the U.S.) and encouraging patient adoption of updated technologies presents significant challenges. Despite its long history and culture significance, traditional medicine faces challenges in terms of standardization, regulation and integration with modern health care systems (70).

## 7 Recent patents in DFUs treatments (year: 2018–2024)

To prepare the review, more than 500 patents related to DFU were overviewed. The patents published in the database of “Google Patent” database were studied from the year 2020-2024. Recently, there has also been an increasing trend in published patents related to diabetic foot ulcers, indicating that this research area is essential in the medical domain. The strategy used for patent literature search by using keywords (1) DFU and (2) year 2018-2024.

The selected patents were thoroughly reviewed and are presented in **Table 9**. **Table 9** collects the patents related to DFU and presents the information under the headings (1) Publication No. (2) Title (3) Inventors (4) Assignees (5) Publication year (6) Citations.

**Table 9:** Selected patents related to Diabetic Foot Ulcer.

Publication Number	Title	Inventors	Assignee	Publication year	Ref.
AU 2023285791 A1	Antimicrobial compounds, compositions, and uses thereof	Kates, Steven A.;Sleet, Randolph B.;Parkinson, Steven	Lakewood Amedex, Inc.	2024	(71)
AU 2023274138 Al	Angiogenesis using stimulated placental stem cells	Kathy E. Karasiewicz-Mendez;AleksandarFrancki;Jeffrey et al.	CelularityInc,	2023	(72)
US20240026417A1	Methods of preparing materials with ammonia oxidizing bacteria and testing materials for ammonia oxidizing bacteria	David R. Whitlock James Heywood SpirosJamas Larry Weiss	Aobiome LLC	2024	(73)
CN117274242A	Wound surface detection method and system based on image recognition	Wang Junying Chen Xiaowei Su Fengmei Mao Jun Yan Zhuo Li Xinting Zheng Xiuzhen Zhang Zhaorao Chen Shouwan .,	Jianyang city people’s hospital	2023	(74)
CN117338699A	Preparation method of rutaecarpahypolye hydrogel and application of rutaecarpahypolye hydrogel in promoting healing of diabetic wound surface	NieXuqiang Mu Xingrui GuRifang .	Zunyi Medical University	2023	(75)
CN117298133A	Application of effective ingredient formula of Guizhi sugar-gangrene in preparation of medicament for treating diabetic foot	Cao Bin Tian Jingzhen Shao Chenglei Zhang Chuanji Liu Yujuan .	Tangning Pharmaceutical Technology Jinan Co ltd	2023	(76)
AU 2023210616 A	Measurement of susceptibility to diabetic foot ulcers	Burns, Martin F.;barringtON, Sara;ross, Graham 0.	BBI Medical Innovations, LLC	2023	(77)
CN116942126A	Intervention device and method for diabetic foot ulcer	Sun Bo Wang Yunqian Zhao Tong	Xian University of Technology	2023	(78)
CN116548926A	Cold stimulation-based diabetic foot screening system and method	Xu Yang Yang Xianjun Sun Yining Wang Hui Zhou Xu Ding Zenghui Gao Lisheng peak Chen Yanyan Sun Shaoming	Zhongke Anhui G60 Intelligent Health Innovation Research Institute)	2017	(79)
20230355520A1	Methods and compositions for treating diabetic foot ulcers	Jeffrey Clark Jeffery King	Pathway Development LLC	2020	(80)
CN116898793A	Lysozyme hydrogel for diabetic foot ulcers and preparation method thereof	Jiang Bangping Shen Xingcan Wang Aihui Li Liqun Liu Xingyu	Guangxi Normal University	2023	(81)
RU2804781C1	Medicinal product for the treatment of ulcer defects in “diabetic foot” syndrome	Evgeniy Vladimirovich Namokonov NadezhdaAnatolyevnaShemyakina ZoyaAleksandrovnaArtamonova et al.	Russia (RU)	2023	(82)
JP2022184911A	Treatment of diabetic foot ulcer using placental stem cells	A. fiskoffsteven ChitkalaDeneshherzberguri Jankovik Vladimir	CelularityInc	2022	(83)
US20230181042A1	Machine learning systems and methods for assessment, healing prediction, and treatment of wounds	Wensheng Fan Jeffrey E. Thatcher PeiranQuan Faliu Yi Kevin Plant Zhicun Gao Jason Dwight	Spectral MD Inc	2023	(80)
CN116726147A	Nursing paste for preventing and treating early diabetic foot as well as preparation method and application thereof	Li Jianquan Wei Xuzhi	Winner Medical Co ltd.	2023	(84)
US20230338296A1	Devices and methods for delivery of oxygen to a wound	Paul MOUNTFORD Mark Borden Robert T. Scribner Robert M. Scribner	Respirogen Inc	2023	(85)
CN220024273U	Special shoes of diabetes foot ulcer treatment stage	Li Yongjie Feng Qiling Mai Lifang Li Xiaomei	Sun Yat Sen Memorial Hospital Sun Yat Sen University	2023	(86)

In this section, the authors portray some selected innovations that primarily focus on DFU healing methods, their medication processes, and other emerging advanced techniques for the prediction of DFU locations. Steven, K et al. (71) published the patent granted to the design of antimicrobial pharmaceutical compound formulas and its application methods for the treatment of an infection of DFU. They also addressed the treatment and administration of the compound to treat other infections, such as lung infection, urinary tract infections, and pneumonia. Subsequently, Kathy E. et al. (72) has also been granted the patent for developing a method of using placental stem cells stimulated by cytokines to promote angiogenesis for the treatment of disorders or diseases resulting from vascularization or poor blood flow. This method demonstrated the use of β-stimulant placental stem cells for the treatment of DFU. Similarly, patent (83) has also been granted as a treatment method for multi-diabetic foot ulcer (DFU) and peripheral arterial disease using CD10+, CD34-, CD105+, and CD200+ placental stem cells. A multidisciplinary approach is crucial for the treatment of ischemic revascularization. In this approach, the inventor used an electromechanical energy-supported device to successfully treat peripheral vascular disease of the limbs caused by DFU. Yuval and Avni (87) have been granted a patent for inventing a device that utilizes pneumatic compression and oscillation energy to treat peripheral circulatory disorders. Additionally, Dolan and O’Donoghue (88) have presented an arrangement in which ultrasonic energy is used to treat blockage of blood vessels due to foot ischemia.

In another patent document (73), utilizing friendly bacteria, the inventor utilized ammonia-oxidizing bacteria (AOB) to treat disorders associated with deficient nitrite levels in the skin or infections caused by pathogenic bacteria. These AOB have the capability of generating nitric oxide with assisted treatment of chronic wounds, such as DFU’s. Furthermore, the usage of modern technology can improve the healing rate and overcome the financial burden on the patients. In this line of advanced technology, image processing for wound-surface detection can be used to improve the healing rate of DFU (74).

It has been established that the elevation of blood glucose in patients with diabetes results in skin stiffness and increased collagen cross-linking. These conditions lead to the damage of blood vessels and muscles in the feet and legs, and an increase in extracellular fluid. Barrington et al. (77) developed an integrated apparatus comprising sensors for DFU treatment. These sensors measure the capacitance value and are used as indicators of the possible locations of the DFUs. In another invention, the electrical impedance (78) and temperature change rate (79) imaging method secured a patent for the detection of the risk of DFU.

An inventor from China highlighted the potential of traditional medicine prepared from herbs. The alkali aqueous patent hydrogel prepared from Rutaecarpine herb (75) showed good results for healing DFU and skin wound surfaces. Similarly, a medicine prepared from Chinese Angelia herb was patented for the treatment of DFU (76). Furthermore, the invention to the field of pharmaceutical preparations, and presented lysozyme hydrogel for diabetic foot ulcers that enhance manganese ions sensitivity to clear residual bacteria and destroy biological membranes (81).

Modern medicinal treatments for foot ulcers have also been continuously explored by researchers. Evgeniy Vladimirovich et al. (82) recommended the medicinal product to treatment of diabetic foot syndrome. The %wt composition of the patent medicine contains sodium selenite 0.15-0.2 Metronidazole 0.25-0.26; dimethyl sulfoxide 20.0-25.0 dissolved in distilled water. They claimed that the composition was anti-allergic and highly effective for the treatment of DFUs.

Several innovative machine learning algorithms for predicting wound healing and associated therapeutic techniques have been presented in numerous published research articles and patents. Machine learning algorithms utilize clinical images for comparison, and thus predict with marginal errors. The technique that acquired medical images from the first wavelength reflected from the damaged tissue regions was patentable to Fan et al. (80) In order to evaluate and forecast healing, the picture pixels were further divided into non-wound and wounded pixels using the ML methodology.

The published patents present several novel methods for the management and treatment of DFUs, which are a valuable source for researchers. Investigations related to DFUs treatment are challenging for researchers. However, a large gap has been observed between research activities and practical applications.

## 8 Conclusions and future outlook

Diabetic foot disease has emerged as a critical research area, necessitating a multidisciplinary approach to address its complex pathophysiology and employing advanced technologies for evaluation. Despite progress in understanding the disease and the available treatment options, further research is needed to identify optimal preventive strategies and portable, low-cost devices for its management. The pathophysiology of diabetic foot ulcers is characterized by the interplay between persistent hyperglycemia and neuropathic, vascular, and immune system components. Effective preventive measures, including patient education, regular foot assessments, and risk stratification, are crucial for managing diabetic foot disease, and these should be implemented in conjunction with a multidisciplinary team’s facilitation of various treatment modalities. The statistical analysis of the correlation between diabetic foot ulcers (DFU) and other complications, such as high blood pressure and diabetic retinopathy, yielded important results but did not demonstrate a significant association with the prediction of DFU occurrence.

The prevalence of diabetes is increasing in underdeveloped countries due to a lack of modern technology. However, the accessibility of knowledge related to diabetic foot ulcer prevention and patient education represents a significant step toward reducing the risk of DFU and improving patients’ quality of life. Advanced tools such as the present AI system, could be important contributors to addressing the growing global challenges presented by the DFU. The integration of nanotechnology, medicine, and artificial intelligence represents a potentially significant approach for developing molecularly tailored nano-medicine and improving overall outcomes for patients with diabetic foot ulcers.

This comprehensive review highlights several effective techniques for managing DFU; however, there is a need to standardize the process of treating foot infections in patients with diabetes. The ethical consideration such as data privacy in technology-based solution (e.g. wearable or tele-medicine platform) should be considered. While the outcomes of employing a multidisciplinary approach to treating DFU are favorable, the potential for low-cost portable devices and cutting-edge technologies to revolutionize medical science should not be overlooked. It is essential to investigate the potential side effects in other organ systems that regulate severe infections when determining the optimal treatment plan for managing glycemic control. Patient self-management or educational camps may help alleviate some of the challenges associated with DFU.
